# Easy-to-configure zero-dimensional valley-chiral modes in a graphene point junction

**DOI:** 10.1126/sciadv.adp6296

**Published:** 2024-09-11

**Authors:** Konstantin Davydov, Xi Zhang, Wei Ren, Matthew Coles, Logan Kline, Bryan Zucker, Kenji Watanabe, Takashi Taniguchi, Ke Wang

**Affiliations:** ^1^ School of Physics and Astronomy, University of Minnesota, Minneapolis, MN 55455, USA.; ^2^ Department of Physics, The Ohio State University, Columbus, OH 43221, USA.; ^3^ Research Center for Electronic and Optical Materials, National Institute for Materials Science, 1-1 Namiki, Tsukuba 305-0044, Japan.; ^4^ Research Center for Materials Nanoarchitectonics, National Institute for Materials Science, 1-1 Namiki, Tsukuba 305-0044, Japan.

## Abstract

The valley degree of freedom in two-dimensional (2D) materials can be manipulated for low-dissipation quantum electronics called valleytronics. At the boundary between two regions of bilayer graphene with different atomic or electrostatic configuration, valley-polarized current has been realized. However, the demanding fabrication and operation requirements limit device reproducibility and scalability toward more advanced valleytronics circuits. We demonstrate a device architecture of a point junction where a valley-chiral 0D PN junction is easily configured, switchable, and capable of carrying valley current with an estimated polarization of ~80%. This work provides a building block in manipulating valley quantum numbers and scalable valleytronics.

## Teaser

In a graphene point junction, switchable valley current with high valley polarization can be easily configured.

## INTRODUCTION

The band edges of typical two-dimensional (2D) semimetals (*1*) and semiconductors are found at two corners of the first Brillouin zone commonly referred to as K and K′ valleys (*2*, *3*). In monolayer transition metal dichalcogenides, dynamic valley polarization can be achieved with optical excitations (*4*–*7*). At a 1D boundary of two bilayer graphene (BLG) domains (natural or gate-defined), topological valley-chiral states can be realized (*8*–*14*) when two critical conditions are simultaneously met: (i) carrier density reaches zero in both the two domains and the boundary, and (ii) the Berry phase (*15*, *16*) of each valley switches sign at the boundary. The valley-chiral 1D state has been experimentally demonstrated in two major device architectures: gate-defined (*8*–*10*, *13*) and naturally occurring (*11*, *12*, *14*, *17*, *18*). The former scheme uses two pairs of split gates perfectly aligned on top of each other, requiring extreme precision in angle and overlay alignment for device fabrication. The charge neutrality in the 1D channel must also be ensured by tuning an additional global gate. The latter scheme relies on natural domains of opposite Bernal-stacking orders. While the device fabrication no longer requires precise overlay alignment, the existence of a natural domain boundary is rare and with its pattern naturally predefined (*11*, *12*, *14*, *19*, *20*), requiring optical identification and limiting versatile circuit design. These successful experiments provide important proof-of-principle demonstration for valleytronics (*8*–*14*, *19*–*29*), but scalability toward the valley-chiral circuit network desires an alternative device scheme with higher yield and simpler fabrication and operation.

## RESULTS

### BLG point junction—A 0D PN junction

**Fig. 1.**
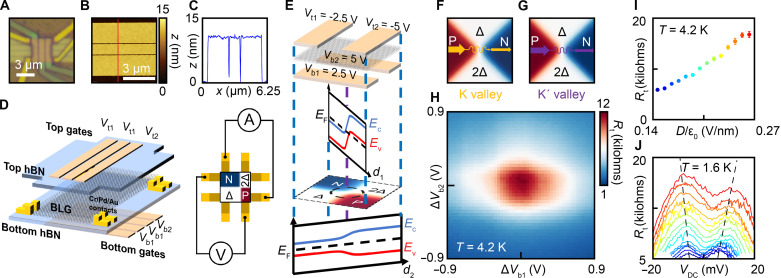
Electrostatically defined zero-dimensional PN junction. (**A**) Optical image of a BLG device with multiple horizontal bottom gates and vertical top gates. (**B**) AFM topography of typical prepatterned bottom gates with (**C**) 1D cut [along the red line in (B)] demonstrating a roughness of less than 1 nm, critical for ballistic electrostatic manipulation. (**D**) Schematics of the device architecture, consisting of multiple top and bottom gates. Gate voltages changes once across an arbitrarily chosen top (bottom) gate separation (**E**) electrostatically dividing the BLG into two N-doped (P-doped) and two insulating (with Δ and 2Δ bandgaps) regions. The top (bottom) gates with the same gate voltage are shown equivalently as a single gate with voltages (*V*_t1_, *V*_t2_, *V*_b1_, *V*_b2_) = (−2.5 V, −5 V, +2.5 V, +5 V). 1D band diagrams along two diagonal directions and the 2D carrier density profile are shown below. The bandgap does not close at the PJ in the center of the device. (**F** and **G**) Carriers from the K and K′ valleys contribute equally to the measured tunneling current across the PJ without valley chirality. (**H**) Measured four-probe resistance of a typical PJ as voltages on the two bottom gates are tuned away from (*V*_b1_ = +2.5 V, *V*_b2_ = +5 V) by Δ*V*_b1_ and Δ*V*_b2_. The resistance peak is observed when a PJ is established at Δ*V*_b1_ = Δ*V*_b2_ = 0, and decreases away from the peak as either of the two insulating regions becomes doped. (**I**) Measured four-probe PJ resistance as a function of the displacement field, and (**J**) the PJ’s resistance as a function DC bias, *V*_DC_, measured at different *D* fields from *D*/ε_0_ = 0.14 V/nm (blue) to *D*/ε_0_ = 0.26 V/nm (red) with an increment of 0.01 V/nm.

Here, we have developed a device architecture of a point junction (PJ), where a PN junction (*30*) and a quantum point contact (QPC) (*27*, *31*–*35*) are simultaneously defined at the center of the device (Fig. 1A). The PJ can be electrostatically configured easily without the need for precise overlay alignment or precise electrostatic tuning. Hexagonal boron nitride (hBN)–encapsulated (*36*) BLG stacks are transferred on top of a series of predeposited bottom gates, each with a 1- to 2-μm width and a ~100-nm gap in between them (Fig. 1, B to D). The hBN thicknesses are deliberately chosen to be equal to or larger than 50 nm (or half of the gates’ separation), to ensure that the electrostatic profile transitions continuously across the graphene region in between adjacent gates, to avoid leaving any region ungated or single-gated. The AFM topography of the local bottom gates displays a roughness of less than 1 nm (Fig. 1, B and C), to minimize lattice strain and remote defects that may cause inter-valley scattering in the graphene above. A series of top gates are subsequently deposited with similar geometry but orthogonal orientation to the bottom gates (Fig. 1D). A pair of 1D edge contacts is then fabricated (*37*) at each of the four corners of the devices, and reactive ion-etching defines the final geometry of the device (Fig. 1A).

We first demonstrate how a PJ can be electrostatically defined. Different gate voltages are applied to the top (bottom) gates, which we denote as (*V*_t1_, *V*_t2_, *V*_b1_, *V*_b2_). The top (bottom) gate voltage value changes once from *V*_t1_ to *V*_t2_ (from *V*_b1_ and *V*_b2_) across an arbitrarily chosen top (bottom) gate separation, electrostatically dividing the BLG into four different regions (of a 2 × 2 grid). The four regions are highly reconfigurable, with each region’s carrier density and gap size (*38*–*40*) determined by a given pair (section S8) of the four gate voltages, and the boundary of the regions determined by the choices of the top (bottom) gate separations across which the gate voltage changes. Figure 1E plots the generalized electrostatic profile of the device, treating top (bottom) gates with the same gate voltage equivalent to as a single gate. For the ease of discussion and intuitive demonstration, we use a device with an equal top and bottom hBN thickness as an example. The carrier type of any of the four regions is given directly by the sign of *V*_t_ + *V*_b_, where *V*_t_ (*V*_b_) is the top (bottom) gate voltage defining the region. At the common corner of the four regions, a trivial (without the valley-chiral modes) PJ can be easily configured by setting *V*_t1_ = −*V*_b1_, and *V*_t2_ = −*V*_b2_, rendering two regions zero carrier density and the other two oppositely doped. For a more general device architecture where the thickness of the top hBN (*d*_t_) and thickness of bottom hBN (*d*_b_) are different, the condition for forming PJ is given by *V*_t1_/*d*_t_ = −*V*_b1_/*d*_b,_ and *V*_t2_/*d*_t_ = −*V*_b2_/*d*_b_. The gate capacitances have been separately characterized for each device studied, consistent with the topographical measurement of hBN used for each device, as well as the voltages at which PJ resistance peaks were observed (section S7).

As an example (Fig. 1E), at (*V*_t1_, *V*_t2_, *V*_b1_, *V*_b2_) = (−2.5 V, −5 V, +2.5 V, +5 V), the top left (bottom right) region of the device is N-doped (P-doped) while the other two regions are gapped (with the same displacement field direction). At the center of the device and the common corner of all four regions, a PN junction (along the direction across P-doped and N-doped regions) and a constriction (QPC, along the direction across two insulating regions) are simultaneously defined—a single point in space where the P and N regions meet to define a 0D PN junction. This is a natural consequence of the electrostatic potential from the device architecture, without requiring elaborate device tuning or precise overlay alignment.

The quantitative details of each region can be seen with a band diagram along two diagonal directions of the device. Between far corners of the P and N regions, the gap size stays the same while the carrier type changes at the PJ. The gap size of the two depleted regions changes at the PJ, but it does not close (the displacement field does not switch sign). We therefore do not expect valley-chiral states in this trivial configuration, i.e., the electrons from both valleys should contribute to the transport equally (Fig. 1, F and G) via nonchiral quantum tunneling. We perform a four-probe measurement of quantum transport in this trivial configuration both as a control study to show whether valley-chiral states (when configured) will make a qualitative difference, and as a characterization of quantum tunneling across the PJ (which exists in a realistic device with a finite barrier height).

To do this, we set the top gates’ voltage to be (*V*_t1_, *V*_t2_) = (−2.5 V, −5 V), while measuring the four-probe resistance (Fig. 1H) from the P to the N side of the device as a 2D sweep of (*V*_b1_, *V*_b2_). The preset top gate voltages, set the target doping and gap size for each region of the eventual PJ (for tunability of each device region). The 2D sweep (instead of requiring three or more parameters) allows us to accurately configure and identify the PJ when measured resistance reaches its maximum value as a consequence of two insulating regions being established simultaneously. The two parameters (Δ*V*_b1_, Δ*V*_b2_) denote the difference between actual voltage applied and the expected value of (*V*_b1_, *V*_b2_) = (+2.5 V, +5 V) at which the PJ is defined with (Δ*V*_b1_, Δ*V*_b2_) = (0, 0) corresponding to the formation of two insulating regions and successful establishment of the PJ. A resistance peak is observed at (Δ*V*_b1_, Δ*V*_b2_) = (0, 0), as expected from the suppression of current via the two regions that become insulating. The size of the displacement field, *D* (and, therefore, the gap), of the two insulating regions when the PJ is formed is chosen to differ by a factor of 2, so that the bulk graphene regions are highly doped and their resistances are comparably negligible; therefore, the measured resistance can be primarily attributed to the tunnel resistance at the PJ. In the remainder of the manuscript, the displacement field, *D*, for a given device configuration is referring to the displacement field of the insulating region with the smaller in magnitude *D* field (while the other insulating region has a *D* value twice in size). In this configuration, the size of the carrier density in the P and N regions, |*n*|, is equal and proportional to the *D* field. The measured four-probe resistance characterizes the quantum tunneling process across the PJ, and monotonically increases with the displacement field in the Δ region (Fig. 1I) as the tunnel barrier at the PJ increases. The resistance’s dependence on DC voltage (Fig. 1J) shows non-ohmic behavior and begins to decrease beyond a 5- to 10-mV bias. This is consistent with the estimated range of gap sizes of Δ = 10 to 20 mV and previously reported values (*39*–*42*) at displacement fields, *D/*ε_0_, ranging from 0.14 to 0.26 V/nm.

### Easy-to-configure 0D valley-chiral modes

**Fig. 2.**
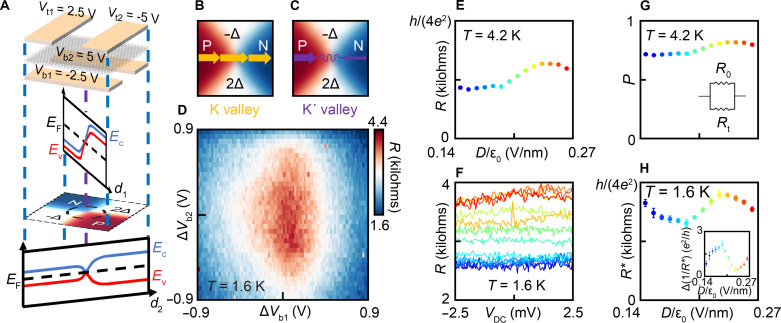
Easy-to-configure zero-dimensional valley valve. (**A**) Electrostatic profile of the device, treating top (bottom) gates with the same gate voltage equivalent to a single gate, at gate voltages (*V*_t1_, *V*_t2_, *V*_b1_, *V*_b2_) = (+2.5 V,−5 V, −2.5 V, +5 V), with 1D band diagrams along two diagonal directions and the 2D carrier density profile shown below. At the common corner of the N-doped (P-doped) and the insulating (with −Δ and 2Δ bandgaps) regions, the bandgap and carrier density flip signs simultaneously, and the PJ is defined satisfying both conditions for valley-chiral modes. (**B** and **C**) While quantum tunneling is expected from both valleys, current from the P to the N region is primarily carried by valley-chiral 1D modes with carriers exclusively from the K valley. (**D**) Qualitative feature of measured four-probe resistance of a typical PJ, as voltages on the two bottom gates are tuned away from (*V*_b1_ = −2.5 V, *V*_b2_ = +5 V) by Δ*V*_b1_ and Δ*V*_b2_. The resistance peak is observed when a valley-chiral PJ is established at Δ*V*_b1_ = Δ*V*_b2_ = 0 and decreases away from the peak as either of the two insulating regions become doped. (**E**) Measured four-probe PJ resistance as a function of the displacement field, *D*, (**F**) and as a function DC bias, *V*_DC_. The measured PJ resistance shows ohmic behavior as expected from ballistic chiral channel and has weaker dependence on the *D* field as the effective barrier for type 2 tunneling vanishes near the PJ. (**G**) Estimated valley polarization and (**H**) net PJ resistance characterized at different *D* fields from *D*/ε_0_ = 0.14 V/nm (blue) to *D*/ε_0_ = 0.26 V/nm (red) with an increment of 0.01 V/nm. The type 2 tunneling in the nontrivial mode can be estimated by the difference (H inset) between the inverse net PJ resistance and 4*e*^2^/*h*.

With the above characterization of trivial tunneling across the PJ, we now add the new component of the valley-chiral modes. This can be done by applying a different gate configuration. An example is shown for (*V*_t1_, *V*_t2_, *V*_b1_, *V*_b2_) = (+2.5 V, −5 V, −2.5 V, +5 V). In contrast to the previous configuration, both the value and the sign of the top (bottom) gate voltage change from *V*_t1_ to *V*_t2_ (from *V*_b1_ and *V*_b2_) across the chosen top (bottom) gate separation. The carrier density and displacement field (and gap) switch sign simultaneously at the PJ (Fig. 2A), satisfying both requirements for valley-chiral states. Similarly, this is automatically ensured by device electrostatics instead of precise tuning and overlay alignment.

If, for example, a current is driven from P to N. While carriers in both K and K′ valleys (Fig. 2, B and C) can contribute via quantum tunneling (as characterized in Fig. 1), only carriers from the K valley (Fig. 2B) can pass through the 0D valley-chiral modes.

Similar to the previous measurement, we preset the top gates’ voltage to be (*V*_t1_, *V*_t2_) = (+2.5 V,–5 V) while measuring the four-probe resistance (Fig. 2D) from the P to the N side of the device as a 2D sweep of (*V*_b1_, *V*_b2_), to identify the gate voltage configuration necessary for PJ formation (zero carrier density and zero *D* field at the PJ). A resistance peak is observed near (Δ*V*_b1_, Δ*V*_b2_) = (0, 0) with respect to the expected (*V*_b1_, *V*_b2_) = (−2.5 V, +5 V). This corresponds to the two insulating regions being simultaneously defined with opposite displacement field (Fig. 2A). The displacement field reaches zero and switches sign at the common corner of the two insulating regions, while the carrier density reaches zero and switches sign at the common corner of the P-doped and N-doped regions (Fig. 2A). The above two locations correspond to the same location of the device, the common corner of all four regions, where the carrier density and displacement field (and gap) reach zero and switch sign simultaneously to define a valley-chiral PJ (Fig. 2A).

The gap sizes of the insulating regions follow the same ratio as the trivial configuration, for a direct control comparison. The resistance itself (Fig. 2E) is substantially smaller than that of the trivial configuration (Fig. 1I) with the same gap sizes (data points with matching color) for the insulating ±Δ regions. Its nearly zero dependence on DC bias (Fig. 2F) suggests ohmic behavior that is expected from a ballistic 1D channel (see section S11 for dependence over larger DC voltage bias range). These observations qualitatively confirm the existence of the valley-chiral modes, naturally defined by the PJ electrostatics, which can be easily fabricated, configured, and tuned.

The measured conductance in this configuration is contributed by two parallel channels: (i) quantum tunneling across the PJ that applies to both valleys via which the current is nonchiral, and (ii) valley-chiral states with a ballistic conductance of 4*e*^2^/*h* and are exclusive to K valley via which the current is valley-chiral. The latter promises 100% valley polarization and 4*e*^2^/*h* quantized conductance while the coexistence of the former results in reduced valley polarization and a measured total PJ conductance higher than 4*e*^2^/*h*. As such, we model the two coexisting mechanisms as conducting channels in parallel from which we estimate a valley polarization of ~80% via the formula (see section S2): *P =* (1 *+ R*/*R*_0_)/2 (Fig. 2G). Despite this limitation on a single PJ, valley polarization can be further improved by operating multiple valley-chiral PJs in series with additional top and bottom gates that are easily scalable (see section S2).

The nonchiral quantum tunneling that compromises the valley polarization can be further distinguished into two types. Type 1: tunnel current via the region directly under/above the local gates away from the PJ where the size of bandgap is constant. This leakage channel can be suppressed by further increasing the bandgap with a larger displacement field. Type 2: tunnel current via the region in between the gate separations in the vicinity of the PJ where the size of the bandgap is determined by the fringing field and has strong spatial dependence that is different in the trivial and nontrivial configurations (see section S3). In the trivial configuration, type 2 tunneling is weaker with a finite tunnel barrier near the PJ, and thus can be further suppressed with a higher *D* field, which is consistent with the comparable rapid monotonic dependence shown in Fig. 1I. In the nontrivial configuration, the tunneling is impossible to eliminate with electrostatics alone as the effective tunnel barrier diminishes near the vicinity of the PJ. As a result, the estimated *P* increases slowly with the displacement field (Fig. 2G) as type 1 tunneling is being suppressed effectively while type 2 is not.

To estimate the type 2 tunneling current, we subtract the conductance of the PJ between when it is configured with and without the valley-chiral state. The size of the gaps and the type 1 tunneling currents is the same in the two configurations. The conductance after subtraction (Fig. 2H) is observed to be 10 to 20% higher than 4*e*^2^/*h* (a value expected if there was no leakage current due to tunneling, which characterizes the type 2 tunneling current near the vicinity of the PJ in the valley-chiral configuration, which is a primary limiting factor for valley polarization).

### Valley-chiral quantum Hall states with topologically protected high polarization

**Fig. 3.**
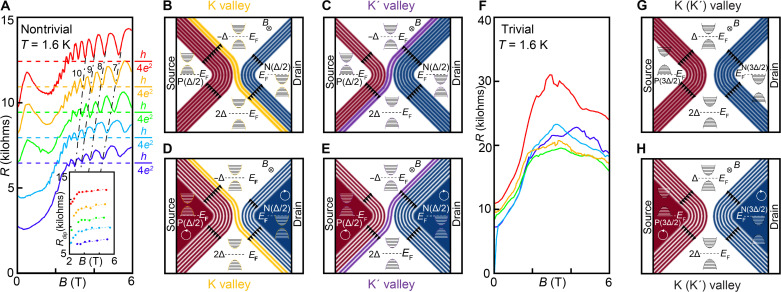
Valley-chiral quantum Hall states with topologically protected high polarization. (**A**) Shubnikov–de Haas oscillations in the resistance of the valley-chiral PJ at different displacement fields from 0.19 V/nm (purple) to 0.23 V/nm (red) with a 0.01 V/nm increment. The plots are offset vertically by 1.5 kilohms and the colored dashed lines refer to *h*/(4*e*^2^)—the expected resistance from valley-chiral states. The dips are found when the Fermi level is in a Landau gap of the bulk P and N regions (dashed line with the corresponding Chern number), consistent with the bulk carrier density at each *D* field. Inset: The oscillation dips’ resistances show a weak *B* field dependence with values around *h*/(4*e*^2^). (**B** to **E**) The spatial distribution of compressible states (red: P type; blue, N type; yellow, zero-energy K valley-chiral; purple: zero-energy K′ valley-chiral) and incompressible states (white). At integer bulk filling, electronic transport occurs exclusively via valley-chiral states both for the K valley (B) and the K′ valley (C), leading to an observed dip resistance of *h*/(4*e*^2^). At partial bulk filling [(D) and (E)], backscattering resistance in the bulk region in series to valley-chiral resistance *h*/(4*e*^2^) gives rise to the observation of resistance peaks. (**F**) Control experiment performed with all four regions defined with a *D* field (in the Δ region) of the same sign (trivial configuration) where the valley-chiral QH states are expected for neither of the two valleys (**G** and **H**). As the ν = 0 Landau gap increases with larger *B* fields, the measured resistance grows sharply and then saturates in contrast to the oscillatory behavior.

While such type 2 tunneling in a valley-chiral PJ cannot be eliminated by electrostatics due to bandgap closing near the PJ, it can be suppressed by a Landau gap at zero charge density (ν = 0) with an out-of-plane magnetic field, *B*, applied (*43*)*.* At low field, the measured resistance of the PJ with and without valley-chiral modes increases with the *B* field as the zero-energy Landau gaps help to suppress both type 1 and type 2 tunneling. At high field, the resistance of the valley-chiral PJ starts to demonstrate Shubnikov–de Haas oscillations (Fig. 3A) with the oscillation minima close to *h*/(4*e*^2^) (dashed lines). Each color plot is taken at different displacement fields offset with equal separation.

The observation is consistent with the expectation from valley-specific Chern numbers in BLG. At *B* = 0 and *D* > 0, while the net Chern number of BLG as a whole is 0 (making it topologically trivial), the K (K′) valley each has an effective Chern number (valley Chern number) of −1 (1) that leads to valley-chiral 1D modes at the PJ. Similarly, at finite magnetic field, Landau quantization gives rise to QH insulators with conventional Chern number *N* (where *N* is defined as the number of electron-type Landau levels below Fermi surface). The valley Chern number can be defined in the K (K′) valley as *C*_K_ = −sign(Δ) + *N* [*C*_K′_ = sign(Δ) + *N*]. The BLG as a whole has its Chern number equal to the Landau level indices, *N*, leading to *N* stripes of conventional QH edge states at the boundary between the doped and undoped regions. The additional edge states at the center of PJ are valley-specific quantum Hall edge states due to the difference in the valley Chern numbers, |*C*_K_–*C*_K′_| = 2, instead of conventional QH states due to different conventional Chern numbers for BLG as a whole (which are zero). The valley-chiral states’ existence is made possible by the PJ’s two insulating regions defined with displacement fields of the opposite sign, instead of simply being transmitted/reflected by the PJ like a conventional QH state at a QPC (*31*, *44*).

When the Fermi level is in gap, BLG has an integrated Berry phase of 2π (−2π) in the K (K′) valleys, totaling a zero Berry phase and a zero (conventional) Chern number, an overall topologically trivial insulator. However, if valley is a good quantum number in the absence of inter-valley scattering, a valley Chern number of −1 (1) can be individually defined within each K (K′) valley. The valley Chern number of each valley switches sign at the boundary between two insulating regions of opposite *D* field, leading to a valley Chern number difference of −2 (2) for the K (K′) valley, giving rise to the valley-chiral modes.

To elaborate on this, we separately illustrate the spatial distribution of compressible states (CS, red: P type, blue: N type, yellow: valley-chiral) and incompressible states (white) for the K valley (Fig. 3, B and D) and the K′ valley (Fig. 3, C and E). At high magnetic field, exactly two QH edge states exist at the PJ with opposite current direction for charges in different valleys (valley-chiral). The existence of these valley-chiral QH states is consistent with a series of resistance dips of *h*/(4*e*^2^) observed when the bulks of both the P and N regions are in a gap and are incompressible (Fig. 3, B and C), whose oscillation periodicity and Chern numbers (Fig. 3A, dashed) are consistent with bulk carrier density at the corresponding *D* fields (section S12). The two valley-chiral states ballistically connect the P and the N side of the device independent of the bulk Landau level indices. When the bulk P and N regions become compressible (Fig. 3, D and E), a resistance peak is measured due to finite resistances of the dissipative compressible bulk region (due to backscattering), in series with the quantized valley-chiral QH states (see section S13).

As a control comparison, we perform the same measurement with the trivial configuration (Fig. 3F) where the two insulating regions and the two doped regions are defined with a *D* field of the same sign. The QH states’ distributions are the same for both valleys (Fig. 3, G and H) because the valley Chern number does not change across any boundary across adjacent device regions, and the valley-chiral QH states are not expected (Fig. 3, G and H). As the ν = 0 Landau gap increases, the tunneling between QH states (*44*, *45*) on the P and N sides becomes more suppressed, leading to a measured resistance that sharply increases with *B* field instead of the oscillatory behavior.

## DISCUSSION

In summary, we have developed a device architecture of a graphene PJ. We demonstrate the transport signature of (i) an electrostatically defined 0D PN junction, (ii) a 0D valley-chiral valve with estimated valley polarization of 80%, and (iii) topologically protected valley-chiral quantum Hall states with ~100% polarization under high magnetic field. All above states can be easily configured without demanding device fabrication protocol and precise electrostatic tuning. The ease of configuration and reproducibility allows valley-chiral valves to be configured in series to further improve the valley polarization, or in networks for valley logic devices (see section S9). The device provides a platform to study valley-polarized quantum phenomena in graphene and a robust device component toward scalable valleytronics.

## MATERIALS AND METHODS

To fabricate the devices, two parallel pieces of 1- to 2-μm-wide metal gates (serving as *V*_b1_, *V*_b2_) with a separation of ~100 nm and consisting of Cr/Pd-Au alloy (1 nm/7 nm) are deposited on a 285-nm-thick SiO_2_ substrate (Fig. 1, B and C). The Pd-Au alloy (40% Pd/60% Au) is chosen to reduce surface roughness compared to the conventional Au deposition. Then, the gates are annealed in a high-vacuum environment at 350°C for 15 min to remove surface residue and to ensure a flat topography. After the annealing, the gates are examined (Fig. 1, B and C) under an atomic force microscope (AFM) confirming their flatness and their lack of contamination. The slight difference in the AFM topography and the target material thickness may arise from (i) the slight miscalibration of the evaporator crystal sensor and/or (ii) the realistic artifact from AFM height measurement across surfaces made of two different materials (SiO_2_ and Pd-Au alloy). However, the device functionality is sensitive only to the roughness of the bottom gate instead of its thickness.

Top hBN, BLG, and bottom hBN flakes (Fig. 1D) were consecutively picked up using polypropylene carbonate (PPC) and polydimethylsiloxane (PDMS) stamps before transferring onto the predeposited gates following the standard dry transfer technique (*36*, *46*). The sample was rinsed in acetone and isopropanol to remove the PPC residue and then annealed in a high-vacuum environment at 350°C for 15 min. AFM topographies of the sample were taken to ensure that it was free of air bubbles and local atomic strain. After that, a pair of parallel metal top gates (serving as *V*_t1_, *V*_t2_) separated by a ~100-nm gap and perpendicular to the bottom gates were fabricated by electron beam lithography and Cr/Pd/Au (1 nm/5 nm/14 nm) deposition. Following that, electrical contacts to gates and ohmic contacts (*37*) to 1D boundaries of the BLG were made by electron beam lithography, dry etching, and subsequent metal deposition (Cr/Pd/Au, 1 nm/5 nm/>120 nm). Last, the final lateral geometry (Fig. 1A) of the device was defined by the ultimate round of dry etching.

The electrical- and magneto-transport measurements for multiple devices studied in this work are performed in a Cryostation s50 (Montana Instruments) at the base temperature of 4.2 K and in a TeslatronPT (Oxford Instruments) at the base temperature of 1.6 K with magnetic fields up to 6 T. The measurements were carried out with AC excitations of 17.777 Hz. The voltage drops and currents were measured using SR830 lock-in amplifiers (Stanford Research Systems) and current preamplifiers (DL: model 1211). The DC bias voltage was controlled by a waveform generator (Keysight: model 33500B). The gate voltage was applied from various voltage sources (Keithley Instruments: model 2400, Yokogawa: model GS200, QDevil: model Q302 QDAC, and a custom-made DAC). Depending on the specific device architecture (number of parallel gates that can be used), the resistance (four-probe for every device except device 4) of one PJ (fig. S1, A and B) and/or two (fig. S1, C and D) PJs in series was measured. In the latter case, we expect the 0D chiral modes to facilitate a 4*e*^2^/*h* conductance across the device (as expected from ballistic quantum conductance) while we expect the nonchiral trivial quantum tunneling resistance, *R*_t_, to be a sum from the tunneling resistance of two PJs (effectively as two resistors in series). In this case, *R*_t_ of a single PJ can be estimated as one-half of the resistance of the two PJs in series.

## Supplementary Materials

### This PDF file includes:

Supplementary Text Figs. S1 to S14 Table S1 References
