# Supplementary material for: Easy-to-configure zero-dimensional valley-chiral modes in a graphene point junction
Source: Sci Adv. 2024 Sep 11;10(37):eadp6296. doi: 10.1126/sciadv.adp6296 (PMC11389794; doi:10.1126/sciadv.adp6296)
Supplement: Supplementary file 1 — Supplementary Text Figs. S1 to S14 Table S1 References [file sciadv.adp6296_sm.pdf]

Science Advances
AAAS

Supplementary Materials for

Easy-to-configure zero-dimensional valley-chiral modes in a graphene point junction

Konstantin Davydov *et al.*

Corresponding author: Ke Wang, kewang@umn.edu

Sci. Adv. **10**, eadp6296 (2024)
DOI: 10.1126/sciadv.adp6296

This PDF file includes:

Supplementary Text
Figs. S1 to S14
Table S1
References

Supplementary Text

S1. Characterization of Type-1 and Type-2 Non-Chiral Tunneling Conductance at Zero *B* Field.

The non-chiral quantum tunneling can be attributed to two types. Type-1: tunnel current via insulating regions directly under/above local gates, away from the PJ, where the size of the bandgap is constant. Type-2: tunnel current via the region in between the gate separation in the vicinity of the PJ. In the "trivial" configuration, the bandgap is non-zero in all four regions of the device. As a result, there will be a finite tunnel barrier (Fig. S1B) between the P and N regions with a height proportional to the bandgap size. Applying a *D* field, thus increasing the bandgap, suppresses both Type-1 and Type-2 tunneling which results in a monotonic growth of R_t as a function of *D* (Fig. 1I).

In contrast, in the valley-chiral configuration, the bandgap always closes at the PJ independent of the *D* field applied. As a result, only Type-1 tunneling can be suppressed (Fig. S1C). The measured resistance of the valley-chiral PJ, *R*, therefore, exhibits a much weaker dependence on *D* that eventually saturates (a higher *D* field no longer helps). At $D/\varepsilon_0 > 0.25$ V/nm, the tunneling resistance continues to (no longer) increases with *D* due to a higher (the absence of) tunnel barrier near the PJ for the "trivial" ("non-trivial") configuration. Together with the slight decrease of the bulk in series resistance of the P and N regions, this leads to a monotonic increase (slight decrease) of the measured PJ resistance as *D* increases beyond $D/\varepsilon_0 >$ 0.25 V for the "trivial" ("non-trivial") configuration.

This qualitative difference in the tunnel barriers near PJ (and Type-2 tunneling) is also responsible for the characterized PJ resistance at zero magnetic field being lower than the expected quantized value of $4e^2/h$. At $B = 0$ and finite *D* field (main manuscript: Fig. 2), the measured conductance ($1/R$) across the PJ with the valley-chiral configuration is composed of two parallel conducting channels (Fig. S2): valley-chiral ballistic 1D states ($1/R_0 = 4e^2/h$), and Type-1 + Type-2 non-chiral tunneling that together contribute to a tunneling conductance of $1/R_t'$. The total measured conductance of the PJ in the "non-trivial" mode can, therefore, be written as $1/R_{non\text{-}trivial} = 1/R = 1/R_0 + 1/R_t'$. In contrast, the conductance of the "trivial" mode is given by $1/R_{trivial} = 1/R_t$. The absence of the $1/R_0$ term is due to the absence the 0D chiral states in the "trivial" configuration, and the tunneling conductance, $1/R_t$, in the "trivial" configuration is smaller than $1/R_t'$ of the same *D* field (but in the "non-trivial" configuration), due to the aforementioned smaller Type-2 tunneling at "trivial" configuration. Therefore, the measured PJ resistance in the "trivial" configuration, $1/R_{trivial} = 1/R_t$, ($< 1/R_t'$), does not characterize the tunneling conductance in the "non-trivial" configuration of the same *D* field, but instead a lower bound of it. The main manuscript shows (Fig. 2H) the net resistance of the valley-chiral PJ by directly finding a difference between the measured conductances of the PJ in the two different configurations, given by $1/R^* = 1/R_{non\text{-}trivial} - 1/R_t = 1/R_{non\text{-}trivial} - 1/R_{trivial}$. The difference between the characterized $1/R^*$ and expected quantized conductance ($1/R_0 = 4e^2/h$), is $\Delta(1/R^*) = 1/R^* - 1/R_0 = 1/R_t' - 1/R_t$, due to the difference in the Type-2 tunneling between the "non-trivial" and "trivial" PJ configurations.

S2. Estimation of Valley Polarization at Zero *B* Field

We use the parallel conducting channel toy-model described in S1 to obtain a rough estimation of the valley polarization of the total current. As elaborated in S1, we assume the ballistic conductance, $1/R_0$, to be valley-chiral and the tunnel conductance, $1/R_t'$, to be non-chiral.

With a bias voltage, V, applied, only carriers from the K valley are allowed across the chiral channel $1/R_0$ while carriers from both valleys contribute equally via non-chiral $1/R_t'$. The total measured current therefore consists of $I_K = V/R_0 + V/(2R_t')$ and $I_{K'} = V/(2R_t')$. The valley polarization can then be estimated by $P = I_K/(I_K + I_{K'}) = (1 + R/R_0)/2$, where we used $1/R = 1/R_0 + 1/R_t'$. Beyond this rough estimation, a direct and accurate characterization of the valley polarization requires a network of four PJs with a different valley chirality with a measurement scheme similar to previous experiments (*8*, *9*) on a gate-defined valley crossroad. Such a device architecture and measurement are future directions beyond the scope of this work.

The maximum characterized valley polarization of a single PJ at zero magnetic field is ~80%, mainly limited by non-chiral tunneling currents. For an ideal device, the valley polarization of a single PJ under zero magnetic field can be further improved (potentially up to 90%), by replacing metal gates with graphite gates, allowing (1) more homogeneous undoped regions that reduce accidental leakage paths near the PJ, and (2) more insulating undoped regions with a larger D field, both of which further suppress Type-1 tunneling according to the simulated barrier profile that sensitively depends on the homogeneity and gap size of the bulk undoped region (SI, section S3). While the valley polarization P of a single PJ at $B = 0$ can never reach 100% (due to the absence of a tunnel barrier at the PJ and presence of Type-2 tunneling), the valley polarization can be further improved to $P^* = 1 - (1 - P)(1 - P) = P(2 - P)$, or by a factor of $P^*/P = 2 - P$, by configuring two PJs in series. This corresponds to a $P^* = 99\%$ for two ideal PJ in series.

S3. Simulation of Tunneling Barriers and Illustration of Type-1 and Type-2 Tunnel Currents

To better understand the qualitative difference between the "trivial" (Fig. S1B) and "non-trivial" (Fig. S1C) configurations of a single PJ, a simulation of the lateral electrostatic configuration in the device is performed in COMSOL Multi-physics. The geometric parameters in the simulation (Fig. S1A), such as the hBN thickness and gates' dimensions are similar to those of Device 1. All four gates in the simulation have the same lateral dimensions, with a length (l) and width (w) of 2075 nm and 1 µm. Fig. S1, B and C show a 500 nm x 500 nm zoomed-in region near the PJ. The two gates in each pair are separated by s = 75 nm with the top gates having height h_t = 20 nm, and the bottom gates having height h_b = 8 nm. The thickness of the top (d_t) and bottom (d_b) hBN layers are set to be equal to 100 nm. The voltages (V_{t1}, V_{t2}, V_{b1}, V_{b2}) applied to the top and bottom gates serve as the boundary conditions, and the chemical potential of BLG is set to be zero (Fermi energy). The out-of-plane electric field D_b (D_t) at BLG due to the top (bottom) gate is simulated by treating the capacitance $\partial n/\partial V_t$ ($\partial n/\partial V_b$) between the top (bottom) gates and the BLG to be constant (effectively modelling the BLG as a metal sheet in COMSOL Multiphysics), in agreement with the experimental characterization of the gate capacitive coupling in the carrier density range used in this study. The charge carrier density in BLG is calculated according to $n = (D_b - D_t)/e$ (Gauss's Law); the displacement field is found from $D = (D_b + D_t)/2$ (per definition of displacement field). The simulated out-of-plane displacement field is then used to calculate the estimated gap size of BLG, E_g, following the well-established and characterized relationship between the two ($dE_g/d|D| \sim 100$ meV·nm/V) (*39*, *42*). The position of the conduction (E_c) and valence (E_v) band edges with respect to the Fermi level (E_F) is calculated according to the carrier density n, the parabolic dispersion relationship with an effective mass m^*=0.039m_e, and the bandgap, E_g. Finally, the effective tunneling barrier height, U_b, at a given position in the sample is estimated as min $\{E_c - E_F$, $E_F -$

$E_v\}$. The horizontal axes in Fig. S1, B and C, indicate the lateral dimensions of the device near the PJ. In Fig. S1, we plot the spatial distribution of charge carrier density (n, indicated by the color) and the tunnel barrier height from the electrostatic simulation. The red and blue areas represent the P-doped and N-doped regions, respectively. The white color domains indicate the insulating regions.

In the "trivial" regime, a finite tunnel barrier is present near the PJ, thus, Type-2 leakage current near the PJ can be suppressed with a larger D field. In the "non-trivial" configuration, Type-2 leakage current near the PJ cannot be eliminated by larger D fields as the bandgap always closes at the PJ. It can only be suppressed at a high magnetic field by establishing a tunnel barrier with the $\nu = 0$ Landau gap.

S4. Electrostatics near Point Junction

To illustrate how the thickness of hBN may affect the electrostatic configuration, we present charge carrier distributions (Fig. S3) modelled in COMSOL Multiphysics for a valley-chiral PJ in two qualitatively different scenarios: (1) the hBNs' thickness being much smaller (Fig. S3A) than the gate separation ($d_t = d_b = 5$ nm); (2) both hBNs' thickness being comparable (Fig. S3B) to the separation ($d_t = d_b = 100$ nm). The charge carrier density and displacement fields in the four bulk regions are set to be same for the two scenarios for direct control comparison. At the limit of thin hBN, the regions between the gate separations are unreached by the fringing fields from the adjacent metal gates. In addition to the four bulk regions, there exist four single-gated regions (whose doping is controlled by a single gate covering the region) and an ungated (undoped) region. The electrostatics yield two PJ in parallel, none of which satisfies the criterion for 0D valley chiral states. In contrast, when hBN thickness is comparable to the gate separation, the expected PJ electrostatics are established, with the carrier density and displacement field uniformly transitioning across the gate separations, and carrier density and displacement field switching sign simultaneously at the PJ, allowing the 0D valley-chiral mode to be established. For this reason, we deliberately chose the hBN with a thicknesses larger than 50 nm (or larger than ½ of the gate separation) to guarantee a continuous electrostatic profile in the vicinity of the PJ, so that any region in between the gate separations are simultaneously tuned by the fringing field from all adjacent gates.

S5. Results from Multiple Devices

Besides the devices (Device 1, Device 2) presented in the main manuscript, transport signatures of both "trivial" and valley-chiral PJs were observed in Device 3-7 (Fig. S4) with the resistances comparable to those in Device 1. Like Device 1, Device 3 was also measured under different displacement fields (Fig. S5). Device 3 reproduced the behavior of Device 1: the resistance of the PJ in the "trivial" regime grew with the displacement field (Fig. S5A) and leveled off in the valley-chiral regime (Fig. S5B).

The resistance of the PJ in the "non-trivial" configuration in Device 5 (Fig. S6A) approaches the expected $h/(4e^2)$ under high magnetic fields, reproducing the main observation in magneto-transport result. Weak signatures of Shubnikov-de Haas oscillations are observed but are ill-defined compared to Device 1. We attribute this to realistic sample-dependent edge profiles. The top gates are used as part of the etching mask when defining the device boundary

with reactive ion etching. Depending on the etch profile, a narrow region of the graphene may be gated ineffectively by the top gate. When conducting compressible strips (QH edge states) move toward the sample boundary at high magnetic fields, this narrow region may create a leakage path (Fig. S6B) around the $\nu = 0$ insulating regions and away from the PJ region.

S6. Estimation of Carrier Mobility.

To calculate the mean free path of the charge carriers in the P-doped region, we estimate the mobility of the charge carriers to be $\mu \sim 50000$ cm^2/(V·s) by fitting the data with the Drude model $\sigma = |n|e\mu + \sigma_0$ (*47–49*), where n is the carrier density, e is the elementary charge, and σ_0 is the conductivity due to residual chemical doping of BLG. The corresponding mean free path (*49*) of the charge carriers is estimated by $l = \frac{h}{2e}\mu\sqrt{|n|/\pi}$ where h is the Planck constant. In the bulk, ~100 nm away from the center PJ, n is on the order of 10^{12} cm^{-2} (with the main data taken close to $n = 3.75 \cdot 10^{12}$ cm^{-2}) and the mean-free path is estimated to be greater than 1 µm, an order of magnitude larger than the characteristic geometric size (~ 100 nm) of the point junction.

S7. Characterization of Gate Capacitive Coupling, Displacement Field, and Charge Carrier Density.

The capacitive coupling between the top and bottom gates with BLG determines the values of the displacement field, D, and charge carrier density, n, inside the PJ. Those within the four bulk regions can be estimated based on a simple parallel plate capacitor model: $D = e[-c_t(V_t - V_{t0}) + c_b(V_b - V_{b0})]/2$; $n = c_t(V_t - V_{t0}) + c_b(V_b - V_{b0})$, where V_t, V_b are the top and bottom gate voltages. V_{t0} and V_{b0} are the top and bottom gate voltage offsets, possibly due to a Schottky barrier or slight intrinsic doping of graphene. The capacitances of the top and bottom gates per unit area are given by $\partial n/\partial V_t = c_t = \varepsilon/(ed_t)$, $\partial n/\partial V_b = c_b = \varepsilon/(ed_b)$, where d_t, d_b are the top and bottom hBN thicknesses (Table 1), $\varepsilon = 3.76\varepsilon_0$, is the hBN permittivity, e is the elementary charge, ε_0 is the vacuum permittivity. The values of V_{t0} and V_{b0} can be found from V_b (V_t) applied to all bottom (top) gates at which the 4-probe resistance reaches a minimum at the charge neutrality point (CNP) (Fig. S7, A, C, E, G). Using the same graph, the ratio between the bottom and top gate capacitances c_b/c_t can be estimated from the slope of the CNP on the V_b-V_t plane, $\tan(\alpha) = \partial V_t/\partial V_b|_{\mathrm{CNP}} = c_b/c_t$ matching the value, $c_b/c_t = d_t/d_b$, calculated from the measured (Table 1) hBN thicknesses. With the calculated capacitances, the dependence of the resistance on n and D can be found (Fig. S7, B, D, F, H) matching the expected behavior from the bandgap by the displacement field. At zero carrier density and $|D/\varepsilon_0| > 0.25$ V/nm, the measured 4-probe resistance reaches ~ 100 kohms at $T = 4.2$ K, typical for dual-gated BLG device with finite gap at zero carrier density.

S8. Versatile Configurations of PJ in a Multi-Gated Device Architecture.

The introduced PJ device architecture with multiple gates has the advantage of configuring a PJ at a flexible location of the device by combining the nearest top (bottom) gates and setting them at the same voltage. As an example, we consider a PJ architecture with three top and three bottom gates where two neighboring top (bottom) gates have the same voltages effectively serving as a single gate. Depending on the choice of the pairs, four different regions (two doped and two insulating) can be established with the boundaries determined by the gates

parked at different voltages (V_{t1}, V_{t2}, V_{b1}, V_{b2}). Thus, both "trivial" (Fig. S8) and valley-chiral (Fig. S9) PJ can be configured at one of the four locations of the device set by the boundaries of the gates across which the gate voltage changes. The adjustable control over the location of PJs gives another degree of freedom in configuring valleytronics circuits toward an easily tunable array of valley filters.

S9. Easy Scaling of PJ Device Architecture toward more Advanced Valleytronics Circuits.

The PJ device architecture can be straightforwardly scaled toward a more advanced valleytronics logic circuit with simply adding more gates to the existing device architecture. As an example, a "crossroad" of four PJs with a different valley chirality can be configured (Fig. S10) with a set of three top gates and three bottom gates. The current from the source contact becomes polarized in the K′ valley after passing the first PJ. The current can then turn left to Drain 1 or turn right to Drain 2 as the chirality of PJs in these two paths remains to be the same (positive displacement field to the left and negative to the right). However, the straight current path (to Drain 3) is forbidden as the PJ in this path allows only electrons in the K valley to pass. This PJ crossroad serves as a basic logic component of the valleytronics circuit, and as a metric to characterize PJs' efficiency in polarizing valley current.

S10. Magneto-Transport under Different Displacement Fields.

The evolution of the magneto-resistance of both a valley-chiral (Fig. S11A) and "trivial" (Fig. 11B) PJ can be observed as a function of the displacement field, *D*. Figure S11 shows the PJ resistance plots as a function of an out-of-plane magnetic field, *B*, for the *D* field increasing from 0.15 to 0.26 V/nm with an increment of 0.01 V/nm (with colors matching those in Fig. 1I and Fig. 2E). The valley-chiral PJ resistances are offset by 1.5 kohms with the dashed lines indicating the valley-chiral states' resistance of $h/(4e^2)$. At the range of the *D* field from 0.19 V/nm to 0.24 V/nm, the leakage current is fully suppressed. The transport is dominated by SdH oscillations of the bulk part, in series with valley-chiral QH states at the PJ, demonstrating a large oscillation amplitude whose dip resistances are close to $h/(4e^2)$. At a low *D* field ($D/\varepsilon_0 <$ 0.19 V/nm), the oscillations start to emerge with a smaller magnitude, consistent with a larger Type-1 leakage current at the low *D* field range (Fig. 1I, Fig. 2E), that cannot be fully suppressed at a finite magnetic field. At high *D* fields of $D/\varepsilon_0 >$ 0.24 V/nm (Fig. S11A, the top three plots), the oscilation amplitude starts to decrease as well. This can be potentially attributed to increased inter-valley scattering at the PJ between the counter-propagating valley-chiral QH states due to a narrower compressible stripe whose width starts to be comparable to the magnetic length at high magnetic fields. In the "trivial" PJ, no valley-chiral states exist, and the magneto-transport is dominated by the tunneling between non-valley-chiral QH states across the $\nu = 0$ Landau gap for the whole range of *D* fields.

S11. Comparison of Bias Dependence of "Trivial" and Valley-Chiral Device Configurations.

At the same applied DC bias voltage, V_{DC}, the current passing through a "trivial" PJ is smaller compared to that in a valley-chiral PJ due to the larger resistance of the former. Thus, a

larger range of V_{DC} can be applied in the "trivial" (Fig. 1J) configuration compared to the valley-chiral one (Fig. 2F) without an additional risk of heating or damaging the sample. To underscore the presence of a finite tunnel barrier at the "trivial" PJ and not the "non-trivial" one, we directly compare the PJ resistance for both cases within the same range of an applied DC bias voltage. Figure S12, A and B, show the dependence of $\Delta R(V_{DC}) = R(V_{DC}) - R(0)$, the change in the PJ resistance, R, as a function of V_{DC}, with respect to both "non-trivial" (Fig. S12A) and "trivial" (Fig. S12B) PJs' resistance at zero DC bias in Device 3. The colors indicate different displacement fields from $D/\varepsilon_0 = 0.16$ V/nm (blue) to $D/\varepsilon_0 = 0.34$ V/nm (red) with a step of 0.03 V/nm. The range of changing ΔR in the "trivial" configuration is almost one order of magnitude larger than that for the valley-chiral PJ indicating the presence of a substantially higher tunnel barrier in the "trivial" case. Moreover, the "trivial" PJ resistance shows non-monotonic non-Ohmic behavior as a function of V_{DC} with peaks at 10 mV $\lesssim |V_{DC}| \lesssim$ 15 mV (corresponding to the tunnel barrier heights) for 0.22 V/nm $\lesssim D/\varepsilon_0 \lesssim$ 0.34 V/nm consistent with the calculated from D bandgaps in the range between 20 mV and 30 mV, twice the barrier heights.

S12. Estimation of Quantum Hall Chern Numbers and Landau Level Degeneracy.

The magnetoresistance demonstrates fluctuating behavior that corresponds to Shubnikov-de Haas (SdH) oscillations of the in-series resistance of the doped bulk. When an integer number, N, of electron-type LLs is filled beneath the Fermi level in the N-doped region, it becomes an incompressible Quantum Hall insulator (with the conventional Chern number defined here as N) with zero bulk resistance in series with the PJ (*50*), leading to an overall measured resistance minimum (dip) in the magneto-transport data (Fig. 3A). The P-doped region has the carrier density of the same size $|n|$, but of the opposite sign, thus it is also dissipationless with the Chern number of $-N$. The dip positions correspond to when the carrier density in the P and N regions, $|n|$, is the integer $|N|$ times the density of states per unit area per LL:

$$|n| = \left(\frac{gB}{\phi_0}\right)|N|, \qquad \text{(S1)}$$

where gB/ϕ_0 is the density of states per unit area per LL (*15*), and ϕ_0 is the flux quantum. To find the number of the filled LLs and estimate their degeneracy, we plot the inverse magnetic field of each dip at $B > 2.5$ T (at which the valley-chiral states are robustly established signified by the dip resistance close to $h/(4e^2)$) as a function of changing in the step of one $|N|$ (Fig. S13). Each plot is offset vertically with an equal separation with the colors representing the displacement fields (that are proportional to $|n|$) matching those in Fig. 3A. Error bars in fitted peak positions primarily arise from the slight D dependence of the background bulk resistance and scan resolution. The plots are consistent with the linear dependence given by equation S1: $1/B = g|N|/(|n|\phi_0)$ (shown as dashed lines in Fig. S13) with $g = 4$, and $|n|$ calculated from the voltage of the corresponding gates and their capacitances (section S7) characterized from the measured hBN thickness. This confirms the observed oscillations are attributed to the SdH oscillations of the bulk P and N regions, with Landau level degeneracy of four, in series with the PJ.

S13. Equilibration of Chemical Potential Between Conventional and Valley-Chiral Quantum Hall States

Figure S14 shows magneto-transport across a valley-chiral PJ at high magnetic field via different quantum Hall states. The contacts are made in the corners of the P and N doped regions. Conventional quantum Hall edges "run through" the contact, while valley-chiral edge states exist

only at the PJ and the selected boundaries between an insulating region and a doped region where the following prerequisite conditions for emergence of valley-chiral states are satisfied: (1) the carrier density reaches zero; (2) valley is a good quantum number with inter-valley scattering suppressed; (3) the displacement field (and, therefore, the integrated Berry phase for each valley) switches sign across the boundary. The condition (2) and (3) are not met for the physical boundaries of the P and N doped regions leading to the contacts. As a result, the current carried by the valley-chiral QH states does not directly come from the contact, instead it comes from equilibrizing its chemical potential with the P and N region, equivalent to P and N region in series with the valley-chiral states. When Landau levels in the P and N region are fully (partially) filled, the average chemical potential of carriers tunneling to the valley-chiral states is equal to (lower than) the source electrical potential, eV_S, effectively corresponding to a zero (non-zero) series resistance of the P and N regions, leading to SdH oscillations' dips (peaks).

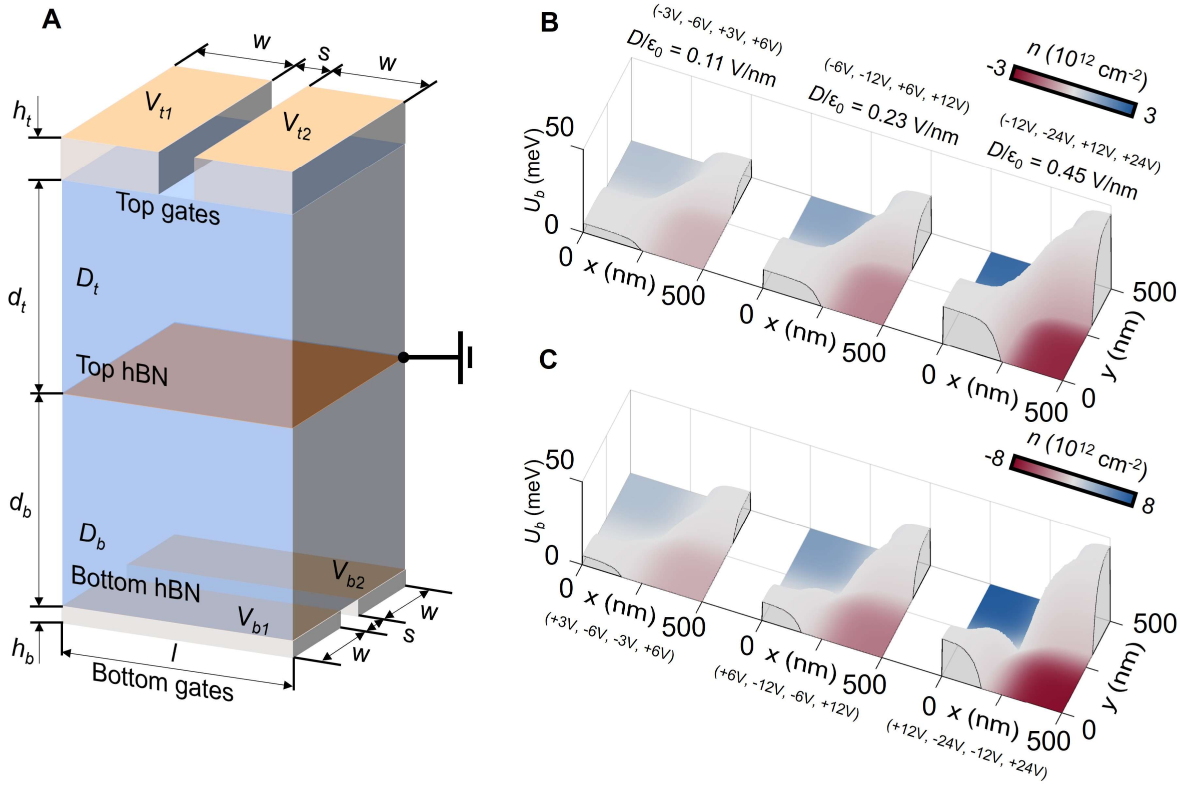

Fig. S1. COMSOL Multiphysics Electrostatics Simulation of Potential Barrier Height in "Trivial" and Valley-Chiral Configurations of PJ.

(**A**) The PJ device geometry in the COMSOL Multiphysics electrostatics simulation with the geometric parameters with all four gates having equal in-plane dimensions l = 2075 nm, w = 1 μm. The heights of the top and bottom gates are h_t = 20 nm and h_b = 8 nm. The top and bottom hBN thicknesses are d_t = 100 nm and d_b = 100 nm. The boundary conditions are set by gate voltages (V_{t1}, V_{t2}, V_{b1}, V_{b2}). The BLG is modelled as a grounded sheet of metal. The displacement fields on top (D_t) and bottom (D_b) of the metal sheet are obtained to calculate the D field inside the BLG. (B) Potential barrier height, U_b, and the charge carrier density (n) near the PJ within the 500 nm x 500 nm region in the "trivial" case at different displacement fields (the values correspond to the displacement field magnitudes in the ±Δ regions) for three different boundary conditions (V_{t1}, V_{t2}, V_{b1}, V_{b2}). (**C**) Same as (B), but for the valley-chiral PJ. Unlike (B), the P and N regions are no longer separated by a finite barrier. The magnitude of the displacement fields in the bulk insulating regions is set to be the same for both configurations.

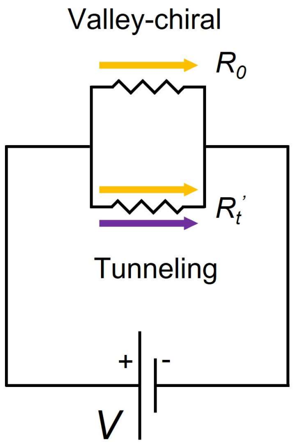

Fig. S2. Two-Channel Toy-Model of Charge Transport in the "Non-Trivial" Case.
The top resistor represents the valley-chiral channel carrying current only from the K valley (yellow arrow); the bottom resistor represents trivial tunneling equally composed of the currents from both the K and K′ valleys (yellow and purple arrows).

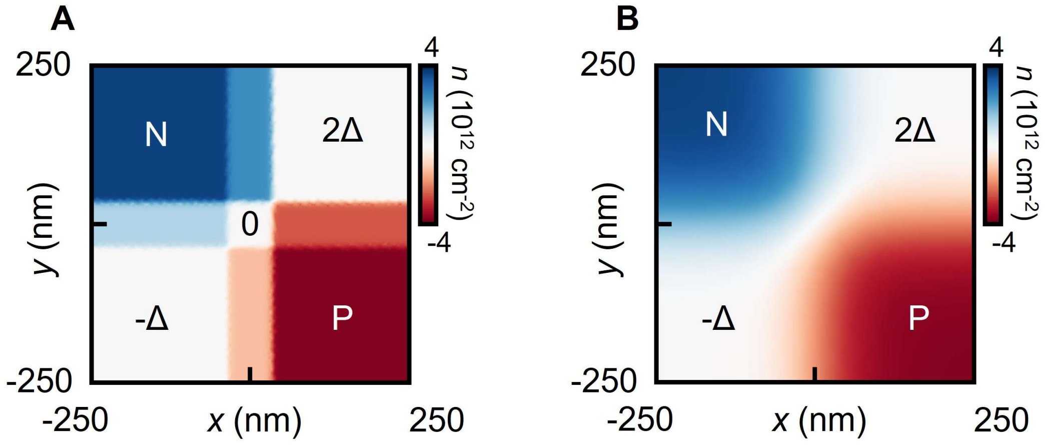

Fig. S3. Electrostatic Configuration in the Vicinity of a Valley-Chiral PJ in the Limits of Thin and Thick hBN Compared to the Gate Separation.

(**A**) Lateral charge carrier density distribution near a valley-chiral PJ with the hBN thickness much smaller than the gate separation. (**B**) Same as (A), but for the hBN thickness comparable to the gate separations. For direct comparison, the charge carrier densities and the displacement fields in the four bulk regions are set to be the same in (A) and (B). In (A), in addition to the four bulk regions, four single-gated (with the doping controlled by a single gate covering the region) and one ungated region (at zero doping) exist resulting in two PJs in parallel, with none of the PJ hosting valley-chiral states. In (B), the charge carrier density and the displacement field smoothly change between the four bulk regions so that the sign of both D and n flips at the center of the PJ enabling 0D valley-chiral states to exist.

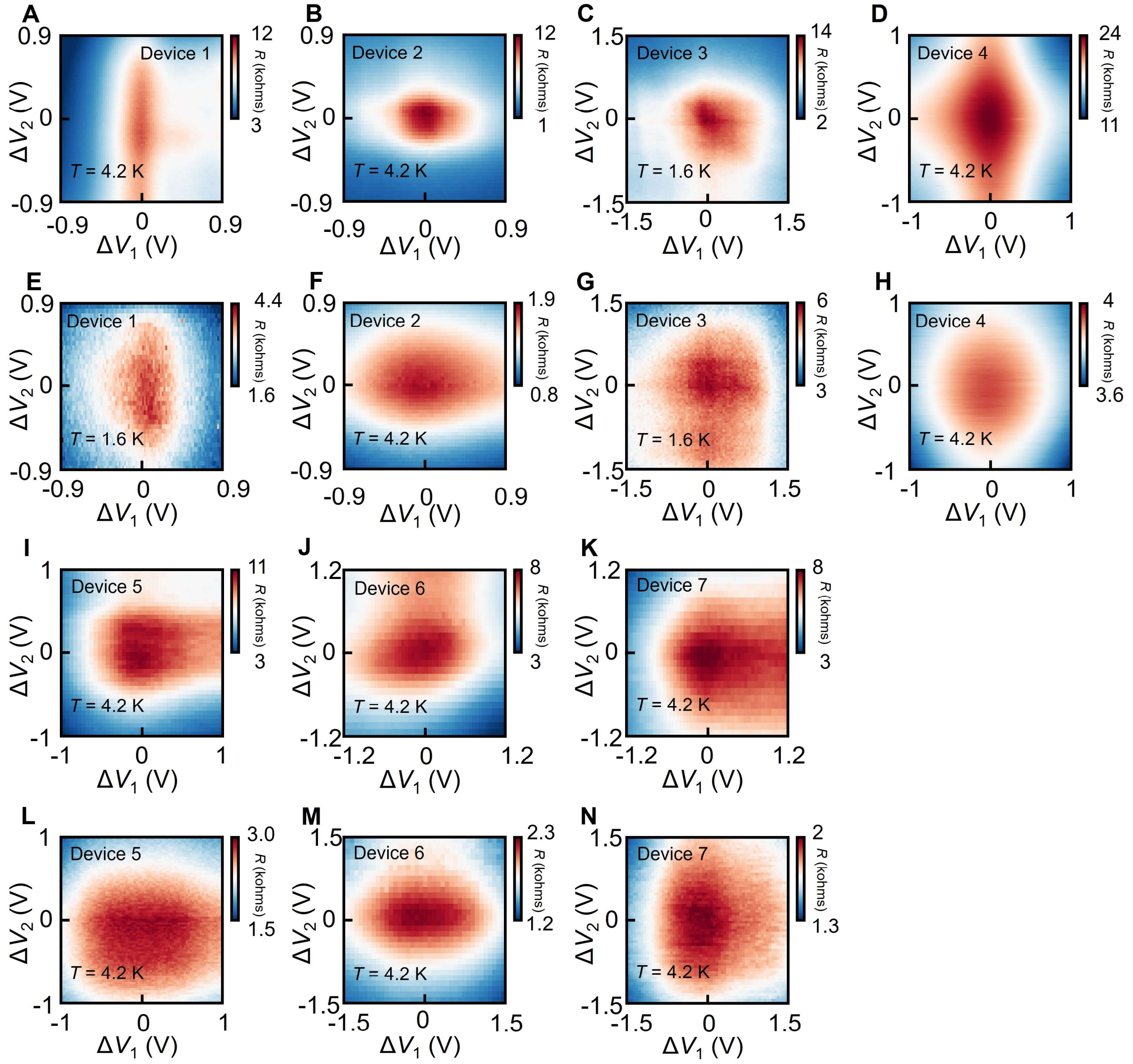

Fig. S4. Transport Signatures of a PJ in Multiple Devices.
A PJ formed in multiple samples (Device 1-7) in both "trivial" (**A**, **B**, **C**, **D**, **I**, **J**, **K**) and "non-trivial" (**E**, **F**, **G**, **H**, **L**, **M**, **N**) cases. In both scenarios, the resistance demonstrates a peak when a PJ is configured. The resistance in the "trivial" case is always notably larger due to the absence of valley-chiral states.

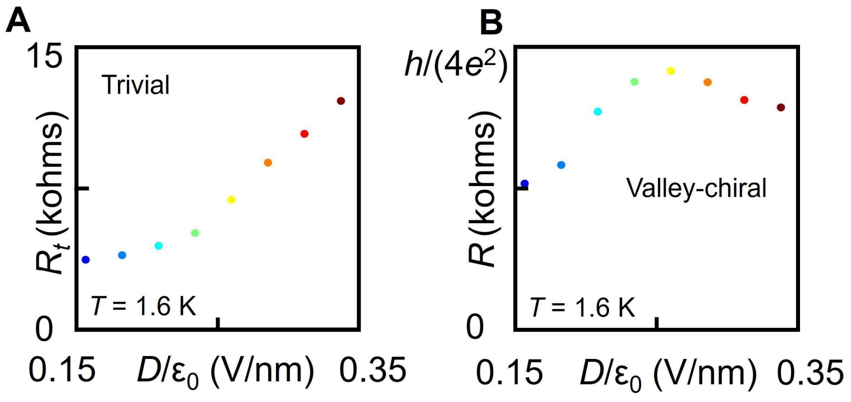

Fig. S5. Displacement Field Dependence of Resistance in Device 3.

(**A**) PJ resistance as a function of displacement field (also indicated by the color) in the Δ region for the "trivial" configuration in Device 3. (**B**) PJ resistance as a function of displacement field in the –Δ region for the "non-trivial" configuration in Device 3.

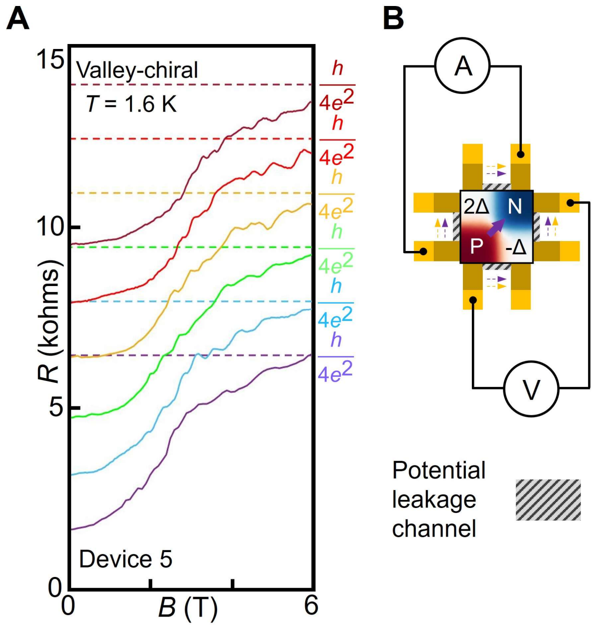

Fig. S6. Magneto-Transport in Valley-Chiral PJ in Device 5.

(**A**) Resistance of the valley-chiral configuration in Device 5 as a function of magnetic field at different D fields in the $-\Delta$ region ranging from 0.15 V/nm (purple) to 0.34 V/nm (dark red) with an increment of 0.04 V/nm. At high magnetic fields, the resistance approaches the predicted resistance, $h/(4e^2)$, of the valley-chiral states which is indicated by the dashed lines. The graphs are vertically offset by 1.5 kohms for clarity. (**B**) Schematics of the device architecture and circuit diagram for Device 5 configured for 4-probe measurements of the resistance. The insulating and doped regions forming a PJ are set up so that the valley-chiral current is carried by the K′ valley (purple arrow) in-gap states. The shaded area indicates the location of potential leakage paths along the etched edges depending on the etch profile that may vary from sample to sample.

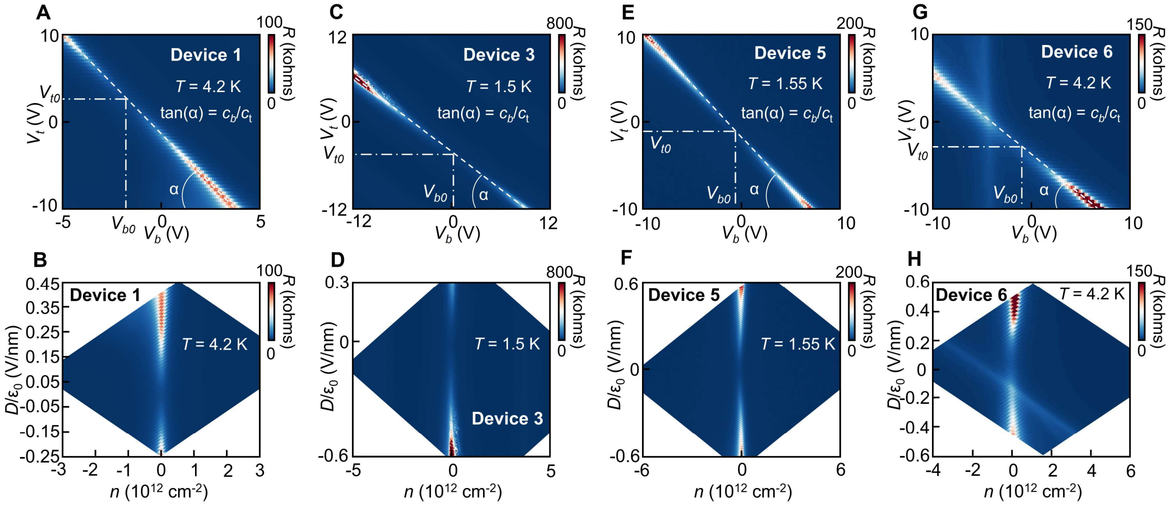

Fig. S7. Gate Capacitive Coupling and Residual Doping Characterization.

(**A**) The resistance of Device 1, R, as a function of all top (V_t) and bottom gates (V_b) voltages. The diagonal line along which the high resistance state is found corresponds to the charge neutrality point (CNP) at carrier density n = 0, the slope of which characterizes the ratio between the bottom ($c_b = \partial n/\partial V_b$) and top ($c_t = \partial n/\partial V_t$) gate capacitances, given by $\tan(\alpha) = \partial V_t / \partial V_b|_{\mathrm{CNP}} = c_b/c_t$. The gate voltage V_{b0}, V_{t0} (dashed) at which the displacement field, D, and carrier density, n, are both zero are identified at the point on CNP with minimum measured R. The value is slightly offset from zero, possibly due to a Schottky barrier or slight intrinsic doping of graphene. (**B**) R as a function of the charge carrier density n and displacement field, D in Device 1. (**C, E, G**) and (**D, F, H**) Same as (A) and (B) but for Device 3, Device 5-6.

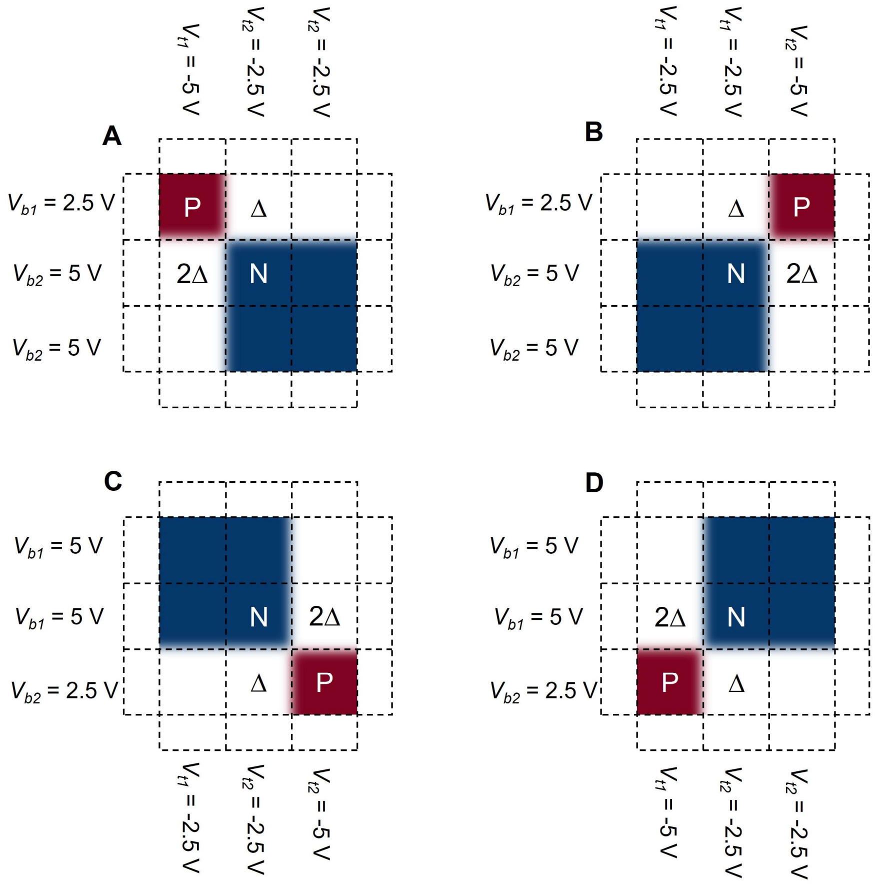

Fig. S8. Electrostatically Tunable Configurations of "Trivial" PJs in a Multi-Gated Device Architecture with Three Top and Three Bottom Gates.

(**A**, **B**, **C**, **D**) Four different locations of "trivial" PJs in a device with three top and three bottom gates (schematically denoted by the dashed lines) at gate voltages (V_{t1}, V_{t2}, V_{b1}, V_{b2}). Two top and two bottom gates in each configuration are at the same voltage effectively acting as a single gate. Depending on the selection of each pair, two P- and N-doped and two insulating regions with the same D field direction (Δ and 2Δ) can establish a "trivial" PJ at for different locations of the device.

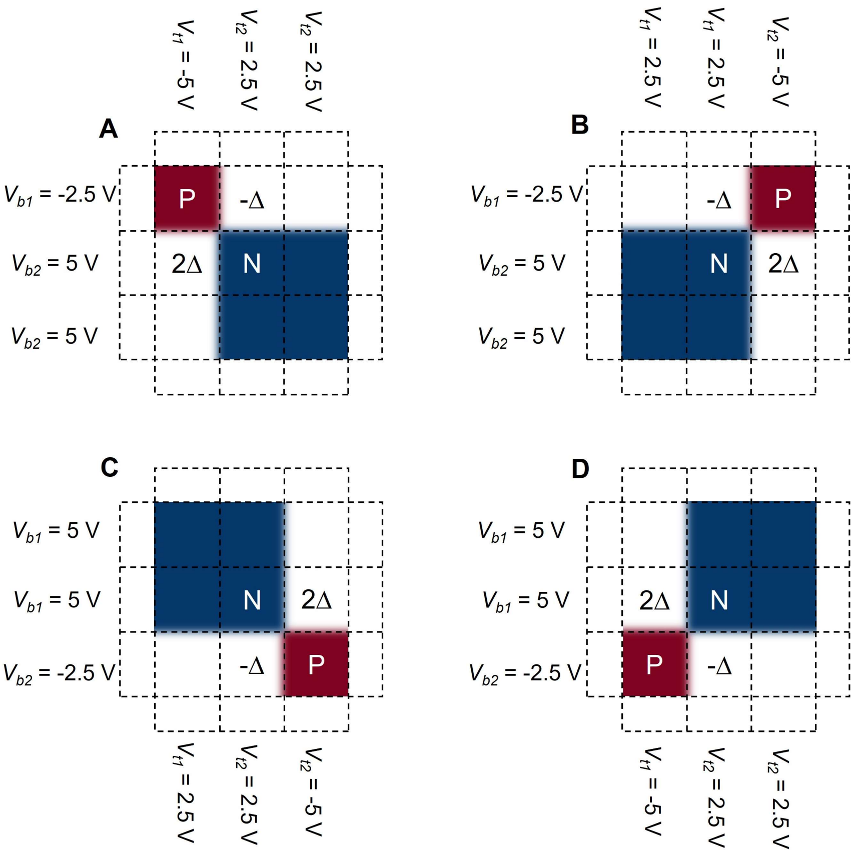

Fig. S9. Electrostatically Defined Locations of Valley-Chiral PJs in a Multi-Gated Device Architecture with Three Top and Three Bottom Gates.

(**A**, **B**, **C**, **D**) Four distinct configurations of valley-chiral PJs in a device with three top and bottom gates (outlined by the dashed lines) at gate voltages (V_{t1}, V_{t2}, V_{b1}, V_{b2}). By setting a particular pair of top and bottom gates to the same voltage, four different locations of two doped (P and N) and two insulating (–Δ and 2Δ) regions can be electrostatically configured defining four different positions of a valley-chiral PJ.

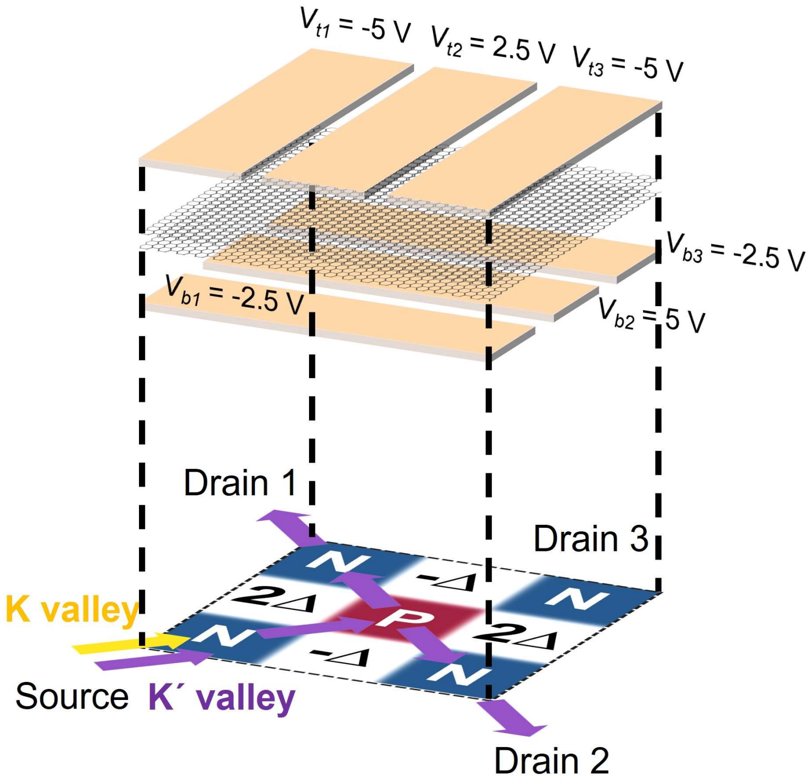

Fig. S10. Array of Four PJs forming a "Crossroad" Device.

Three top and three bottom gates at the voltages (V_{t1}, V_{t2}, V_{t3}) = (–5V, +2.5V, –5V) and (V_{b1}, V_{b2}, V_{b3}) = (–2.5V, +5V, –2.5V) defining four valley-chiral PJs composing a "crossroad" device with four contacts in the corners. By passing the first PJ, the initially valley unpolarized current from the source becomes K´ valley polarized. After that, the K´ polarized current can flow to Drain 1 and Drain 2, passing through the two PJs with the same chirality as the first one (positive D field on the left and negative on the right with respect to the current's direction). The PJ in front of Drain 3 has the opposite chirality passing only K valley electrons and thus blocking the current to Drain 3.

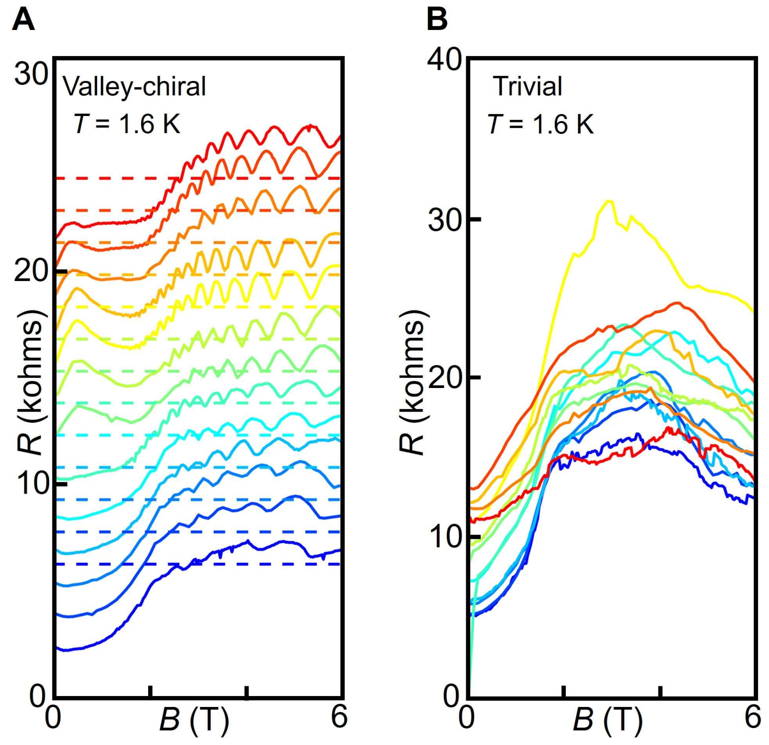

Fig. S11. Displacement Field Dependence of Magneto-Transport through PJ at Full Range of Displacement Field *D*.

(**A**) Valley-chiral PJ resistance as a function of *B* at *D* fields increasing from 0.15 V/nm (blue) to 0.26 V/nm (red) with a step of 0.01 V/nm. The plots are vertically displaced by 1.5 kohms with the colored dashed lines indicating the valley-chiral states resistance, $h/(4e^2)$, approached by SdH oscillations' dips. (**B**) Magnetic field dependence of the resistance of a "trivial" PJ for the same color coded set of *D* fields as in (A) with no vertical offset. In contrast to (A), the almost one order of magnitude larger PJ resistance for the entire range of *D* fields primarily stems from the tunneling between non-valley-chiral QH states across the $\nu = 0$ Landau gap at sufficiently large magnetic fields.

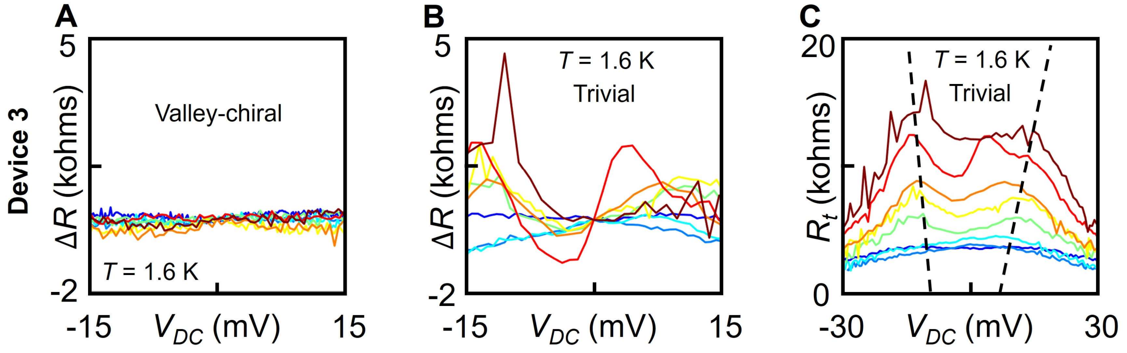

Fig. S12. Direct Comparison of DC Voltage Bias Dependence of Valley-Chiral and "Trivial" PJ Resistance in Device 3.

(**A**) The change, $\Delta R(V_{DC}) = R(V_{DC}) - R(0)$, in the valley-chiral PJ resistance, R, with respect to the zero DC bias baseline as a function of V_{DC} in Device 3. (**B**) Same as (A) but for the "trivial" PJ. For direct comparison, the DC voltage bias ranges match in the "trivial" and "non-trivial" configurations. (**C**) Non-Ohmic behavior of the "trivial" PJ resistance in Device 3 for an extended range of V_{DC} with the resistance peaks at different displacement fields (marked by the colors and ranging from D/ε_0 = 0.16 V/nm (blue) to D/ε_0 = 0.34 V/nm (red) with a step of 0.03 V/nm) highlighted by the dashed lines.

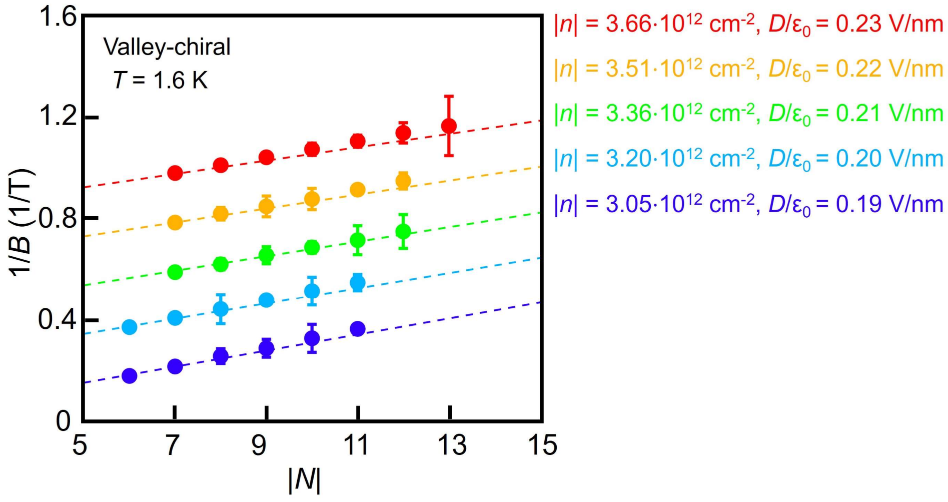

Fig. S13. Estimation of Filled Landau Level Indices and Landau Level Degeneracy from SdH Dip Magnetic Fields in Device 1.

The measured dip positions (dots, in inverse magnetic field $1/B$) and calculated (dashed) dip positions from Landau levels with degeneracy $g = 4$ at the corresponding carrier density $|n|$ of the bulk P and N regions. The quantitative agreement confirms that the observed oscillations are attributed to the SdH oscillations of the bulk P and N regions, with 4-fold degenerate Landau levels, in series with the PJ.

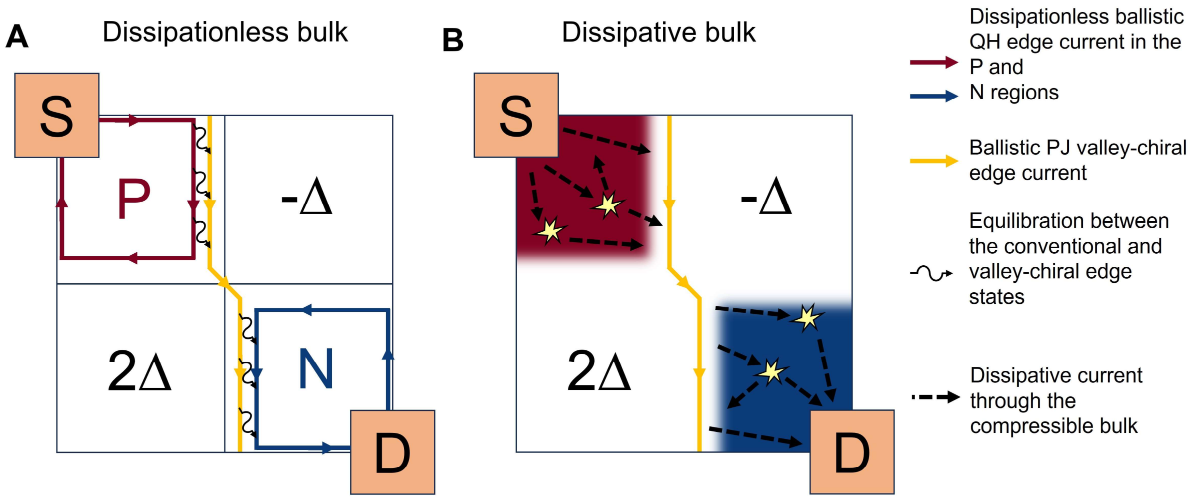

Fig. S14. Equilibration of Point Junction Valley-Chiral Edge Current with Contacts via Dissipationless and Dissipative Transport through Doped Regions.

The valley-chiral states exist continuously across the vertical boundary of the four regions via the PJ, but are not defined at the physical edges of the P and N doped region. (**A**) When the bulk P- and N- doped regions are in a Landau gap and are incompressible, carriers are injected from the source contact to the P-type conventional dissipationless QH edge states (red), which then equilibrize with the valley-chiral QH states (yellow), before equilibrizing with the N-type conventional dissipationless QH edge states connected to the drain. (**B**) When the bulk P and N regions have Landau levels that are partially filled and the bulk becomes compressible, carriers need to undergo dissipative non-ballistic transport, either to reach ballistic valley-chiral QH states from the source, or to reach the drain from the valley-chiral QH states. In either scenario, when Landau levels in the P and N regions are fully (partially) filled, the average chemical potential of the carriers tunneling to the valley-chiral states is equal to (lower than) the source electrical potential, eV_S, effectively corresponding to a zero (non-zero) series resistance of the P and N regions, leading to SdH oscillations' dips (peaks).

Device Number	1	2	3	4	5	6	7
d_t (nm)	115	60	70	100	60	62	62
d_b (nm)	50	50	75	100	50	65	65

Table S1. Top (d_t) and Bottom (d_b) hBN Thicknesses in Measured Devices.

REFERENCES AND NOTES

1. A. K. Geim, Graphene: Status and prospects. *Science* **324**, 1530–1534 (2009).

2. K. S. Novoselov, A. K. Geim, S. V. Morozov, D. Jiang, Y. Zhang, S. V. Dubonos, I. V. Grigorieva, A. A. Firsov, Electric field effect in atomically thin carbon films. *Science* **306**, 666–669 (2004).

3. K. S. Novoselov, A. K. Geim, S. V. Morozov, D. Jiang, M. I. Katsnelson, I. V. Grigorieva, S. V. Dubonos, A. A. Firsov, Two-dimensional gas of massless Dirac fermions in graphene. *Nature* **438**, 197–200 (2005).

4. G. Aivazian, Z. Gong, A. M. Jones, R.-L. Chu, J. Yan, D. G. Mandrus, C. Zhang, D. Cobden, W. Yao, X. Xu, Magnetic control of valley pseudospin in monolayer WSe2. *Nat.Phys.* **11**, 148–152 (2015).

5. Y. Li, J. Ludwig, T. Low, A. Chernikov, X. Cui, G. Arefe, Y. D. Kim, A. M. van der Zande, A. Rigosi, H. M. Hill, S. H. Kim, J. Hone, Z. Li, D. Smirnov, T. F. Heinz, Valley splitting and polarization by the Zeeman effect in monolayer $MoSe_2$. *Phys. Rev. Lett.* **113**, 266804 (2014).

6. K. F. Mak, K. L. McGill, J. Park, P. L. McEuen, The valley Hall effect in MoS_2 transistors. *Science* **344**, 1489–1492 (2014).

7. H. Zeng, J. Dai, W. Yao, D. Xiao, X. Cui, Valley polarization in MoS_2 monolayers by optical pumping. *Nat. Nanotechnol.* **7**, 490–493 (2012).

8. J. Li, R.-X. Zhang, Z. Yin, J. Zhang, K. Watanabe, T. Taniguchi, C. Liu, J. Zhu, A valley valve and electron beam splitter. *Science* **362**, 1149–1152 (2018).

9. H. Chen, P. Zhou, J. Liu, J. Qiao, B. Oezyilmaz, J. Martin, Gate controlled valley polarizer in bilayer graphene. *Nat. Commun.* **11**, 1202 (2020).

10. J. Li, K. Wang, K. J. McFaul, Z. Zern, Y. Ren, K. Watanabe, T. Taniguchi, Z. Qiao, J. Zhu, Gate-controlled topological conducting channels in bilayer graphene. *Nat. Nanotechnol.* **11**, 1060–1065 (2016).

11. L.-J. Yin, H. Jiang, J.-B. Qiao, L. He, Direct imaging of topological edge states at a bilayer graphene domain wall. *Nat. Commun.* **7**, 11760 (2016).

12. L. Ju, Z. Shi, N. Nair, Y. Lv, C. Jin, J. Velasco, C. Ojeda-Aristizabal, H. A. Bechtel, M. C. Martin, A. Zettl, J. Analytis, F. Wang, Topological valley transport at bilayer graphene domain walls. *Nature* **520**, 650–655 (2015).

13. J. Lee, K. Watanabe, T. Taniguchi, H.-J. Lee, Realisation of topological zero-energy mode in bilayer graphene in zero magnetic field. *Sci. Rep.* **7**, 6466 (2017).

14. F. R. Geisenhof, F. Winterer, A. M. Seiler, J. Lenz, I. Martin, R. T. Weitz, Interplay between topological valley and quantum Hall edge transport. *Nat. Commun.* **13**, 4187 (2022).

15. K. S. Novoselov, E. McCann, S. V. Morozov, V. I. Fal'ko, M. I. Katsnelson, U. Zeitler, D. Jiang, F. Schedin, A. K. Geim, Unconventional quantum Hall effect and Berry's phase of 2π in bilayer graphene. *Nat. Phys.* **2**, 177–180 (2006).

16. Y. Zhang, Y.-W. Tan, H. L. Stormer, P. Kim, Experimental observation of the quantum Hall effect and Berry's phase in graphene. *Nature* **438**, 201–204 (2005).

17. L. Jiang, Z. Shi, B. Zeng, S. Wang, J.-H. Kang, T. Joshi, C. Jin, L. Ju, J. Kim, T. Lyu, Y.-R. Shen, M. Crommie, H.-J. Gao, F. Wang, Soliton-dependent plasmon reflection at bilayer graphene domain walls. *Nat. Mater.* **15**, 840–844 (2016).

18. F. R. Geisenhof, F. Winterer, S. Wakolbinger, T. D. Gokus, Y. C. Durmaz, D. Priesack, J. Lenz, F. Keilmann, K. Watanabe, T. Taniguchi, R. Guerrero-Avilés, M. Pelc, A. Ayuela, R. T. Weitz, Anisotropic strain-induced soliton movement changes stacking order and band structure of graphene multilayers: Implications for charge transport. *ACS Appl. Nano Mater.* **2**, 6067–6075 (2019).

19. Z. Qiao, J. Jung, Q. Niu, A. H. MacDonald, Electronic highways in bilayer graphene. *Nano Lett.* **11**, 3453–3459 (2011).

20. A. Rycerz, J. Tworzydło, C. W. J. Beenakker, Valley filter and valley valve in graphene. *Nat. Phys.* **3**, 172–175 (2007).

21. I. Martin, Y. M. Blanter, A. F. Morpurgo, Topological confinement in bilayer graphene. *Phys. Rev. Lett.* **100**, 036804 (2008).

22. D. Xiao, W. Yao, Q. Niu, Valley-contrasting physics in graphene: Magnetic moment and topological transport. *Phys. Rev. Lett.* **99**, 236809 (2007).

23. Z. Qiao, J. Jung, C. Lin, Y. Ren, A. H. MacDonald, Q. Niu, Current partition at topological channel intersections. *Phys. Rev. Lett.* **112**, 206601 (2014).

24. R. V. Gorbachev, J. C. W. Song, G. L. Yu, A. V. Kretinin, F. Withers, Y. Cao, A. Mishchenko, I. V. Grigorieva, K. S. Novoselov, L. S. Levitov, A. K. Geim, Detecting topological currents in graphene superlattices. *Science* **346**, 448–451 (2014).

25. Y.-M. Lin, V. Perebeinos, Z. Chen, P. Avouris, Electrical observation of subband formation in graphene nanoribbons. *Phys. Rev. B* **78**, 161409 (2008).

26. J. L. Garcia-Pomar, A. Cortijo, M. Nieto-Vesperinas, Fully valley-polarized electron beams in graphene. *Phys. Rev. Lett.* **100**, 236801 (2008).

27. K. Sakanashi, N. Wada, K. Murase, K. Oto, G.-H. Kim, K. Watanabe, T. Taniguchi, J. P. Bird, D. K. Ferry, N. Aoki, Valley polarized conductance quantization in bilayer graphene narrow quantum point contact. *Appl. Phys. Lett.* **118**, 263102 (2021).

28. Y. Shimazaki, M. Yamamoto, I. V. Borzenets, K. Watanabe, T. Taniguchi, S. Tarucha, Generation and detection of pure valley current by electrically induced Berry curvature in bilayer graphene. *Nat. Phys.* **11**, 1032–1036 (2015).

29. M. Sui, G. Chen, L. Ma, W.-Y. Shan, D. Tian, K. Watanabe, T. Taniguchi, X. Jin, W. Yao, D. Xiao, Y. Zhang, Gate-tunable topological valley transport in bilayer graphene. *Nat. Phys.* **11**, 1027–1031 (2015).

30. X. Zhang, W. Ren, E. Bell, Z. Zhu, K.-T. Tsai, Y. Luo, K. Watanabe, T. Taniguchi, E. Kaxiras, M. Luskin, K. Wang, Gate-tunable Veselago interference in a bipolar graphene microcavity. *Nat. Commun.* **13**, 6711 (2022).

31. B. J. van Wees, H. van Houten, C. W. J. Beenakker, J. G. Williamson, L. P. Kouwenhoven, D. van der Marel, C. T. Foxon, Quantized conductance of point contacts in a two-dimensional electron gas. *Phys. Rev. Lett.* **60**, 848–850 (1988).

32. L. A. Cohen, N. L. Samuelson, T. Wang, K. Klocke, C. C. Reeves, T. Taniguchi, K. Watanabe, S. Vijay, M. P. Zaletel, A. F. Young, Spontaneous localization at a potential saddle point from edge state reconstruction in a quantum Hall point contact. arXiv:2401.10433 [cond-mat.mes-hall] (2024).

33. J. Ingla-Aynés, A. L. R. Manesco, T. S. Ghiasi, K. Watanabe, T. Taniguchi, H. S. J. van der Zant, Ballistic electron source with magnetically controlled valley polarization in bilayer graphene. *Phys. Rev. Lett.* (2024); https://journals.aps.org/prl/accepted/a5071Y03Yb71cc84510d4141c2753f31bd49da233.

34. L. A. Cohen, N. L. Samuelson, T. Wang, K. Klocke, C. C. Reeves, T. Taniguchi, K. Watanabe, S. Vijay, M. P. Zaletel, A. F. Young, Nanoscale electrostatic control in ultraclean van der Waals heterostructures by local anodic oxidation of graphite gates. *Nat. Phys.* **19**, 1502–1508 (2023).

35. L. A. Cohen, N. L. Samuelson, T. Wang, T. Taniguchi, K. Watanabe, M. P. Zaletel, A. F. Young, Universal chiral Luttinger liquid behavior in a graphene fractional quantum Hall point contact. *Science* **382**, 542–547 (2023).

36. C. R. Dean, A. F. Young, I. Meric, C. Lee, L. Wang, S. Sorgenfrei, K. Watanabe, T. Taniguchi, P. Kim, K. L. Shepard, Boron nitride substrates for high-quality graphene electronics. *Nat. Nanotechnol.* **5**, 722–726 (2010).

37. L. Wang, I. Meric, P. Y. Huang, Q. Gao, Y. Gao, H. Tran, T. Taniguchi, K. Watanabe, L. M. Campos, D. A. Muller, One-dimensional electrical contact to a two-dimensional material. *Science* **342**, 614–617 (2013).

38. E. V. Castro, K. S. Novoselov, S. V. Morozov, N. M. R. Peres, J. M. B. L. dos Santos, J. Nilsson, F. Guinea, A. K. Geim, A. H. C. Neto, Biased bilayer graphene: Semiconductor with a gap tunable by the electric field effect. *Phys. Rev. Lett.* **99**, 216802 (2007).

39. Y. Zhang, T.-T. Tang, C. Girit, Z. Hao, M. C. Martin, A. Zettl, M. F. Crommie, Y. R. Shen, F. Wang, Direct observation of a widely tunable bandgap in bilayer graphene. *Nature* **459**, 820–823 (2009).

40. E. Icking, L. Banszerus, F. Wörtche, F. Volmer, P. Schmidt, C. Steiner, S. Engels, J. Hesselmann, M. Goldsche, K. Watanabe, T. Taniguchi, C. Volk, B. Beschoten, C. Stampfer, Transport spectroscopy of ultraclean tunable band gaps in bilayer graphene. *Adv. Electron. Mater.* **8**, 2200510 (2022).

41. K. F. Mak, C. H. Lui, J. Shan, T. F. Heinz, Observation of an electric-field-induced band gap in bilayer graphene by infrared spectroscopy. *Phys. Rev. Lett.* **102**, 256405 (2009).

42. T. Taychatanapat, P. Jarillo-Herrero, Electronic transport in dual-gated bilayer graphene at large displacement fields. *Phys. Rev. Lett.* **105**, 166601 (2010).

43. K. Wang, T. Hou, Y. Ren, Z. Qiao, Enhanced robustness of zero-line modes in graphene via magnetic field. *Front. Phys.* **14**, 23501 (2019).

44. W. Ren, X. Zhang, J. Ma, X. Han, K. Watanabe, T. Taniguchi, K. Wang, Selective manipulation and tunneling spectroscopy of broken-symmetry quantum Hall states in a hybrid-edge quantum point contact. *Phys. Rev. B* **108**, 245423 (2023).

45. K. Wang, A. Harzheim, T. Taniguchi, K. Watanabei, J. U. Lee, P. Kim, Tunneling spectroscopy of quantum Hall states in bilayer graphene $p-n$ junctions. *Phys. Rev. Lett.* **122**, 146801 (2019).

46. A. K. Geim, I. V. Grigorieva, Van der Waals heterostructures. *Nature* **499**, 419–425 (2013).

47. L. Banszerus, M. Schmitz, S. Engels, J. Dauber, M. Oellers, F. Haupt, K. Watanabe, T. Taniguchi, B. Beschoten, C. Stampfer, Ultrahigh-mobility graphene devices from chemical vapor deposition on reusable copper. *Sci. Adv.* **1**, e1500222 (2015).

48. K. I. Bolotin, K. J. Sikes, J. Hone, H. L. Stormer, P. Kim, Temperature-dependent transport in suspended graphene. *Phys. Rev. Lett.* **101**, 096802 (2008).

49. A. S. Mayorov, R. V. Gorbachev, S. V. Morozov, L. Britnell, R. Jalil, L. A. Ponomarenko, P. Blake, K. S. Novoselov, K. Watanabe, T. Taniguchi, A. K. Geim, Micrometer-scale ballistic transport in encapsulated graphene at room temperature. *Nano Letters*. **11**, 2396–2399 (2011).

50. K. v. Klitzing, G. Dorda, M. Pepper, New method for high-accuracy determination of the fine-structure constant based on quantized Hall resistance. *Phys. Rev. Lett.* **45**, 494–497 (1980).
